# Supplementary material for: COVID-19 related knowledge, attitudes, and practices in Indian Population: An online national cross-sectional survey
Source: PLoS One. 2022 Mar 3;17(3):e0264752. doi: 10.1371/journal.pone.0264752 (PMC8893705; doi:10.1371/journal.pone.0264752)
Supplement: S1 Appendix — (DOCX) [file pone.0264752.s001.docx]

**Supplementary 1 (S1) Appendix**

Knowledge, Attitude, and Practice Questionnaire on Coronavirus disease (Covid-19)

**General and Demographic assessment**

**1. How old are you? Please specify your age in completed years………………………………**

**2. What is your gender?**

1. Male

2. Female

3. other

3. **How many people live in your household (who share a common kitchen) including yourself?** ……………………………………………………………..

**(I) Number of children <5 years of age (below 5 year)** ...................

**(II) Elderly people >60 years of age (above 60)** ............................................

**4. Which amongst the following best describes you (Your profession)?** (Please tick any one option)

1. Unemployed
2. Housewife
3. Student
4. Unskilled labourer
5. Skilled labourer
6. Private job
7. Government Job
8. Business
9. Healthcare worker
10. Others, please mention …………….

**5. What is the highest level of education you have completed?**

1. No school (uneducated)

2. Elementary school (till class 5)

3. High school (till class 10)

4. College (Graduation)

5. Higher education (post-graduate or above)

6. Others, please mention …………….

**6. What is your monthly family income (In Rupees)?.**...............**...**

**7.** **City/Village and state where you live currently, please mention** …………………..

**8.** The a**rea you live in belongs to which category**

1. Urban
2. Semi-urban
3. Town
4. Rural

**9. How far do you live from the nearest health clinic or hospital?** *(Select only one Answer*

1. 0–10 kilometers
2. 11–20 kilometers
3. 21–30 kilometers
4. 30 -50 kilometers
5. Above 50 kilometers
6. **Have you heard of coronavirus? *(Select only one Answer)***
7. Yes
8. No
9. Don’t know
10. No answer
11. **Have you or any member of your family got infected with Coronavirus?** *(Select only one Answer*
    1. Yes
    2. No
    3. Don’t know
    4. No answer

**Section- 1 Knowledge and Awareness**

1. **Do you know about the reason for the current lockdown**? *(Select only one Answer)*
2. Yes, Please Mention…………
3. NO
4. Do not know
5. **When did you first hear about Coronavirus?** *(Select only one Answer)*
6. Many years ago
7. Last year
8. In the last few months (2-4)
9. In the last few weeks (2-4)
10. In the last few days (2-4)
11. Today
12. No answer
13. Other ……………………
14. **Where/from whom did you first hear about Coronavirus?** (*Select multiple answers*)
15. Family members
16. Relatives
17. Friends
18. Neighbours
19. Community meeting
20. Government announcement
21. Newspapers and magazines
22. Radio
23. Social Media (WhatsApp, Facebook etc)
24. TV
25. Brochures, posters and other printed materials
26. Health workers at the health center
27. Religious leaders
28. Teachers
29. Other (please explain):
30. No answer
31. **Do you know anybody who has Coronavirus Disease (Covid-19) in your locality?**

*(Select only one Answer)*

1. Yes
2. No
3. Do not know
4. No answer
5. **If you or somebody in your household had a mild/high fever now, what might be wrong with him?** (*Select multiple answers*)
6. Seasonal fever
7. Viral fever
8. Vector-borne disease (Dengue, chikungunya, Malaria, Kala-azar, Japanese Encephalitis etc.)
9. Coronavirus disease
10. Cannot say
11. No answer
12. Other (Please mention) ……………………
13. **The presence of which of the following features will hint you about having a coronavirus disease?** (*Select multiple answers*)
14. Fever
15. Headache
16. Rash
17. Cough
18. Tiredness
19. Nausea
20. Cough that lasts longer than three weeks
21. Joint pain
22. Chest pain
23. Vomiting
24. Conjunctivitis (red eyes)
25. Diarrhoea
26. Haemorrhage/bleeding
27. Shortness of breath
28. No answer
29. Other ……………………
30. **Does everybody who gets Coronavirus infection show symptoms?**  *(Select only one Answer)*
31. Yes
32. No
33. Maybe
34. Do not know
35. **As per the best current knowledge, how can a person get Coronavirus?** (*Select multiple answers*)
36. Mosquito bite
37. Drinking polluted water
38. Through sexual intercourse
39. Touching surfaces or objects in a public place
40. Through shaking hand
41. Through animal contacts
42. Through hugging people
43. Through coughing and sneezing (e.g. airborne)
44. From breast milk
45. From a blood transfusion
46. From a dirty environment
47. Don’t know
48. Other (please mention) ……………
49. **For maximum how long the Coronavirus can survive outside the human body on any surface or object?**  *(Select only one Answer)*
50. 3 hours
51. 8 hours
52. 24 hours
53. Three days
54. More than four days
55. Cannot say
56. Do not know
57. Can coronavirus be prevented*? (Select only one Answer)*
58. Yes
59. No
60. May be
61. Do not know
62. **How can a person prevent him(her)self from getting Coronavirus infection?** *(Select multiple Answer)*
63. Avoid shaking hands while greeting
64. Covering mouth and nose properly when coughing or sneezing
65. Washing hands properly after touching items in public places
66. Maintain Social Distancing
67. Closing windows at home
68. Using mosquito repellent or spray on your body
69. Wear full body covering clothes
70. Covering mouth with a mask
71. Abstain from sexual intercourse
72. Keeping clean household environment
73. Using mosquito net
74. Using alcohol-based sanitizers on hands
75. Through good nutrition
76. By praying
77. Do not know
78. Other (please mention): ……
79. **What is the range of social distancing?** *(Select only one Answer)*
80. 5 feet-10 feet
81. 6 feet-12 feet
82. 12 feet-20 feet
83. Did not hear the social distancing word
84. Do not know
85. Other (please mention): ……
86. **Which kind of people should be quarantined during current Coronavirus disease (Covid-19) transmission?** *(Select multiple Answer)*
    - 1. Did not listen the word Quarantine
      2. Heard the word, but don’t know the meaning
      3. Infected Individual
      4. exposed Individual
      5. healthy Individual
      6. Risk Individual
      7. don’t know
      8. Other (Please mention):
87. **What is the time period of quarantine in Coronavirus disease (Covid-19)?** *(Select only one Answer)*
88. 7 days
89. 10 days
90. 14 days
91. 15 days
92. 20 days
93. Don’t know
94. **Is there treatment available for Coronavirus disease (Covid-19)?** *(Select only one Answer)*
95. Yes
96. No
97. May be
98. No answer
99. **What should one do if s/he gets Coronavirus disease (Covid-19)?** *(Select multiple Answer)*
100. Have plenty of rest
101. Take medicine to lower the fever and reduce pain
102. Drink a lot of fluids and take healthy diets
103. Seek emergency treatment in Hospital
104. There is no treatment available
105. As advised by your family doctor
106. No answer
107. Other (Please mention) ……………….
108. **Who can get infected with Coronavirus?** *(Select multiple Answer)*
109. Adult Males
110. Adult Females
111. Elderly person / person of geriatric age or above 60 years
112. Children below five years of age
113. Health workers
114. Anybody can get Coronavirus disease (Covid-19)
115. People with pre-existing medical conditions (such as asthma, diabetes, heart disease)
116. People with disabilities
117. Poor people (poverty-stricken)
118. Traveler
119. No answer
120. Other (Please mention) ………………
121. **Whom do you trust the most to give you accurate information about Coronavirus disease (Covid-19)?** *(Select multiple Answer)*
122. Family
123. Friends or neighbours
124. Local representatives (RWA, Gram Pradhan)
125. Local healer / traditional healer/ faith Healer (Quack, Neem-Hakeem, Ojha)
126. Religious / Spiritual leader
127. ASHA/Midwife (ANM) of your Village or Locality
128. Health workers at the health center
129. Private doctor
130. Television
131. Posters
132. Newspapers
133. Internet
134. Social media (WhatsApp, Facebook etc.)
135. National Government Organization (Ministry of Health and family welfare, Govt of India, ICMR)
136. International organizations (WHO, CDC)
137. No answer
138. Other (Please mention) ……………….
139. **What is the current coronavirus pandemic called?** *(Select only one Answer)*
140. SARS
141. Coronavirus
142. SARS-CoV-2
143. Covid19
144. No answer
145. Other (Please mention) …………
146. **Do you think there is a link between SARS, MERS and Covid-19?** *(Select only one Answer)*
147. Yes
148. No
149. May be
150. Do not know

**Section-2 Attitude**

1. **Do you think that Coronavirus disease (Covid-19) can create a serious issue/problem in your community?** *(Select only one Answer)*
2. Yes
3. No
4. Maybe
5. No answer
6. **Do you think the current measure of nationwide lockdown is enough to control the spread of Coronavirus disease (Covid-19)**? (*Select only one Answer)*
7. Yes
8. No
9. Maybe
10. No answer
11. **What worries or concerns you most about Coronavirus disease (Covid-19)?**  *(Select only one Answer)*
12. It can kill you or member of your family
13. It can harm pregnant women and the unborn baby in your family
14. It can permanently disable you or member of your family
15. It can make you or member of your family sick
16. It can kill people in your community
17. It can disturb your economic condition
18. No answer
19. Other (please mention) ………………
20. **How much do you think is** **the risk that you or your family members will get Coronavirus disease (Covid-19) in the next 1-2 months?**

*(Select only one Answer)*

1. High risk
2. Medium risk
3. Low risk
4. No risk
5. Already have a covid-19 patient in home
6. No answer
7. **Whose responsibility is it to prevent you / your household / your community from getting Coronavirus disease (Covid-19)?** (*Select multiple answer*)
8. Personal responsibility (individual)
9. Household head (head of the family)
10. Community Leader / religious leaders
11. Local healer / traditional healer/ faith Healer (Quack, *Neem-Hakeem, Ojha*)
12. Health workers
13. Local district administration
14. National government
15. State government
16. International organizations (WHO)
17. God
18. No answer
19. Other (specify)…………….
20. **If you came in contact of a Coronavirus disease (Covid-19) suspected / affected patient, what would you do?** (*Select multiple answer*)
21. Inform family member
22. Inform local representative (Gram Pradhan, RWA etc.)
23. Go to hospital yourself
24. Inform the local authority
25. Get tested from Laboratory
26. Go to self-isolation
27. Go for self-quarantine
28. Wait for symptoms for confirmation
29. Will not tell anyone
30. No answer
31. Other (please mention) ………………….
32. **If you suspect that you have coronavirus disease, who would you talk to about your condition?**

(*Select multiple answer*)

1. The doctor or another medical worker

2. Spouse

3. Parent

4. Children

5. Other family members

6. Close friend

7. No one

8. Other (please mention) ………………….

1. **Which is the best available cure for coronavirus disease?** *(Select only one Answer)*
2. Herbal remedies/Ayurveda remedies
3. Home rest without medicine
4. Praying
5. Faith Healing (*Jhad-fook*)
6. Allopathy (English) based medicine and Hospitalization
7. Yunani based treatment
8. Homeopathic based treatment
9. Yoga practicing
10. Do not know
11. Other (please mention) ………………….
12. **Who can effectively treat a person with Coronavirus disease (Covid-19)?** *(Select only one Answer)*
13. Public hospital/ Government Hospital
14. Local Healer (Quack, Neem-Hakeem)
15. Private Hospital
16. Do not know
17. Other (specify)………
18. **If somebody in your family gets Coronavirus disease (Covid-19), you would want it to remain private /a secret.**  *(Select only one Answer)*
19. Strongly agree
20. Agree
21. Disagree
22. Strongly disagree
23. No answer
24. **If a person gets Coronavirus disease (Covid-19), should they have discriminated against or stigmatized because of it?*)*** *(Select only one Answer)*
25. Yes
26. No
27. Maybe
28. Don’t know
29. No answer
30. **In your opinion, who is responsible for the transmission of Coronavirus disease (Covid-19) in your community/region?........................**
31. **Do you think you have enough information about Coronavirus disease (Covid-19)?**

*(Select only one Answer)*

1. Yes
2. No
3. Maybe
4. No answer
5. **If you need to know about coronavirus disease, what would you like more information about?** *(Select Multiple Answer)*
6. Cause
7. Signs and symptoms
8. Prevention
9. Treatment options
10. Government action
11. Consequences of having a virus
12. Isolation/Quarantine
13. I have enough information
14. No answer
15. Others (Please mention) …………………

**Section-3 Practices**

1. **Since you heard about Coronavirus disease (Covid-19), what action have you taken to prevent yourself / your household from getting Coronavirus disease (Covid-19)**? (*Select multiple answer*)
2. Wore covering clothes and gloves
3. Used mask
4. Covered mouth with a simple cloth
5. Sprayed or fumigated my home
6. Washed hand for at least 20 seconds daily with soap
7. Used sanitizer
8. Avoided handshake and hugging
9. Avoided touching objects at public places
10. Avoided going outside of the home
11. Prayed to God
12. No action is taken as I am not at risk
13. No answer
14. Other (please mention) …………….
15. **What challenges/difficulties did you face in taking that action?**

(*Select multiple answer*)

1. Difficult to take time from my routine to take preventive measures
2. Difficult to find money and resources to take preventive measures
3. Difficult to have access to necessary items (e.g. to buy mask or sanitizer)
4. I had to overcome people around me who did not want me to take action
5. I did not face any challenges or difficulties in taking action
6. Not applicable as I did not take any action
7. No answer
8. Other (please mention) ……………….
9. **If you have not taken any action to prevent yourself from getting Coronavirus disease (Covid-19), what is the reason?**  (*Select multiple answer*)
10. Not applicable as I have already taken such measures
11. I am not at risk / my household is not at risk
12. I do not go outside of my house
13. I do not think Coronavirus disease (Covid-19) is a problem
14. I do not mind if I get Coronavirus disease (Covid-19)
15. Preventing Coronavirus disease (Covid-19) is not a priority for me
16. I do not have the time to take prevention measures
17. I do not have the resources or access to preventative measures
18. I do not think preventative measures are effective
19. I do not know how to prevent getting Coronavirus disease (Covid-19)
20. Other people are doing what is necessary to prevent me from getting Coronavirus disease (Covid-19)
21. You cannot prevent getting Coronavirus disease (Covid-19)
22. No answer
23. Others (specify)……………..
24. **what action have you taken to prevent your community from getting Coronavirus disease (Covid-19)?** (*Select multiple answer*)
25. Donated money to the government
26. Donated money to other organizations
27. Sanitized the building or locality
28. Stop going outside
29. Created awareness
30. Prayed to god
31. Feed the needy people
32. Stopped people for going outside
33. Did not do anything
34. No answer
35. Other (Please mention) ………………
36. **Which of the following has acted to protect you / your household / your community from getting Coronavirus disease (Covid-19)?***)* (*Select multiple answer*)
37. District Administration/ Local Administration
38. Municipal Corporation
39. RWA/Gram Pradhan/ Panchayat
40. NGO
41. Police
42. Community people
43. Spiritual Leaders
44. Others…………………….
45. **Before the Coronavirus disease (Covid-19) pandemic occurred, which of the following did you routinely do?** (*Select multiple answer*)
46. Washed hands regularly
47. Used sanitizer on your hands frequently
48. Used face masks regularly
49. Avoided going outside unnecessary
50. Avoided handshakes
51. None of the above
52. Other (Please mention) ……………
53. **As per your best knowledge,** **what are the top three most effective ways of preventing Coronavirus disease (Covid-19) or infection?**

*[3 answers]*

1…………………..

2……………….

3………………….

1. **In the last 30 days, have you or any member of your family experienced fever for more than 3 days?** *(Select only one Answer)*
2. Yes
3. No
4. Maybe
5. Don’t know
6. No answer
7. **Would you like to test yourself for Coronavirus infection?** *(Select only one Answer)*
8. Yes
9. No
10. Maybe
11. No answer
12. **If there will be a vaccine available against Coronavirus disease (Covid-19), would you consider having it?** *(Select only one Answer)*
13. Yes
14. No
15. Maybe
16. No answer
17. **In the last 30 days, have you or any member of your family member visited a Doctor or Hospital for seeking care?** *(Select only one Answer)*
18. Yes
19. No
20. Maybe
21. No answer
